# Intraoperative detection of second inferior vena cava during para‐aortic lymphadenectomy for advanced‐staged ovarian cancer: Lessons to be learned

**DOI:** 10.1002/ccr3.2965

**Published:** 2020-06-08

**Authors:** Stamatios Petousis, Chrysoula Margioula‐Siarkou, Beatriz Navarro, George Mavromatidis, Konstantinos Dinas, Frederic Guyon

**Affiliations:** ^1^ Gynaecologic Oncology Unit Institut Bergonie Bordeaux France; ^2^ 2nd Department of Obstetrics and Gynaecology Aristotle University of Thessaloniki Thessaloniki Greece

**Keywords:** left renal vein, ovarian cancer, para‐aortic lymphadenectomy, preoperative imaging, Second inferior vena cava

## Abstract

As the presence of second inferior vena cava may alter the extent of para‐aortic lymphadenectomy, early preoperative imaging diagnosis is of great significance to avoid intraoperative difficulties.

## Graphical abstract

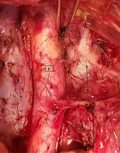

## 1 INTRODUCTION

The presence of second (additional) vena cava is a described yet rare anatomic variation.^1^, ^2^ As such a variation may alter the level of left renal vein which is the upper limit of para‐aortic lymphadenectomy in advanced‐stage ovarian cancer, it worth highlights the significance of preoperative acknowledge of such an alteration in order to achieve uncomplicated completion of para‐aortic lymphadenectomy.

## 2 CLINICAL IMAGE PRESENTATION

**Figure 1:**
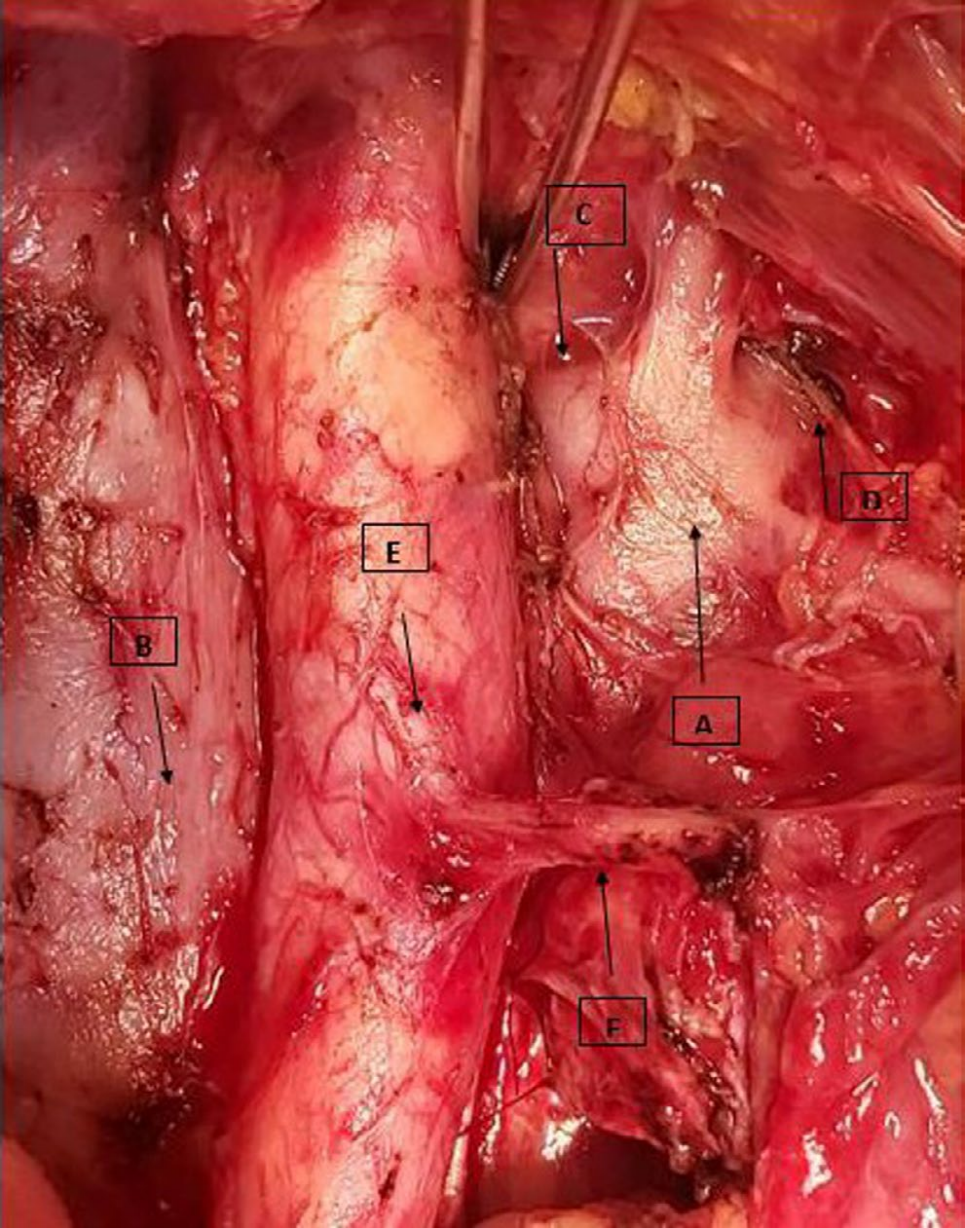
Second inferior vena cava randomly discovered during para‐aortic lymphadenectomy

A 61‐year‐old patient was admitted for interval debulking after neoadjuvant chemotherapy for high‐grade serous ovarian cancer. Preoperative imaging indicated the presence of bulky nodes, therefore necessitating para‐aortic lymphadenectomy, while posed no suspicion for anatomic variations regarding inferior vena cava. The inability of finding left renal vein, which presents the upper surgical limit, posed the suspicion of its potential posterior origin from inferior vena cava. Cautious preparation of tissues leads finally to the discovery of an additional inferior vena cava from which left renal vein was originated, much lower that the anticipated surgical limit (Figure 1). Persistence of surgical preparation permitted the successful completion of para‐aortic lymphadenectomy, which was extended lower than initially expected. Cautious preoperative imaging evaluation of great vessels is of remarkable significance in order to optimize surgery and avoid unnecessary extension of lymphadenectomy that may lead to severe intraoperative complications.

## CONFLICT OF INTEREST

None declared.

## AUTHOR CONTRIBUTIONS

Stamatios Petousis, Chrysoula Margioula‐Siarkou, and Frederic Guyon wrote the initial draft. Stamatios Petousis and Beatriz Navarro have edited the clinical image. Stamatios Petousis, Chrysoula Margioula‐Siarkou, Beatriz Navarro, and Frederic Guyon performed the surgical operation. George Mavromatidis and Konstantinos Dinas significantly revised the draft.

## References

[1] Chiarugi M , Fregoli L , Iacconi P . Inferior vena cava duplication. Updates Surg. 2015;67:325‐327. 26022427 10.1007/s13304-015-0301-8

[2] Mazengenya P . Multiple variations of the renal and testicular vessels: possible embryological basis and clinical importance. Surg Radiol Anat. 2016;38:729‐733. 26507071 10.1007/s00276-015-1584-7

